# Swelling of Ti_3_C_2_T_x_ MXene in Water and Methanol at Extreme Pressure Conditions

**DOI:** 10.1002/advs.202307067

**Published:** 2023-12-14

**Authors:** Artem Iakunkov, Ulrich Lienert, Jinhua Sun, Alexandr V. Talyzin

**Affiliations:** ^1^ Department of Physics Umeå University Umeå SE‐901 87 Sweden; ^2^ DESY Photon Science 22607 Hamburg Germany; ^3^ Department of Industrial and Materials Science Chalmers University of Technology Göteborg SE‐412 96 Sweden

**Keywords:** 2D materials, compressibility, high pressure, MXene, swelling

## Abstract

Pressure‐induced swelling has been reported earlier for several hydrophilic layered materials. MXene Ti3C2Tx is also a hydrophilic layered material composed by 2D sheets but so far pressure‐induced swelling is reported for this material only under conditions of shear stress at MPa pressures. Here, high‐pressure experiments are performed with MXenes prepared by two methods known to provide “clay‐like” materials. MXene synthesized by etching MAX phase with HCl+LiF demonstrates the effect of pressure‐induced swelling at 0.2 GPa with the insertion of additional water layer. The transition is incomplete with two swollen phases (ambient with d(001) = 16.7Å and pressure‐induced with d(001) = 19.2Å at 0.2 GPa) co‐existing up to the pressure point of water solidification. Therefore, the swelling transition corresponds to change from two‐layer water intercalation (2L‐phase) to a never previously observed three‐layer water intercalation (3L‐phase) of MXene. Experiments with MXene prepared by LiCl+HF etching have not revealed pressure‐induced swelling in liquid water. Both MXenes also show no anomalous compressibility in liquid methanol. The presence of pressure‐induced swelling only in one of the MXenes indicates that the HCl+LiF synthesis method is likely to result in higher abundance of hydrophilic functional groups terminating 2D titanium carbide.

## Graphical abstract

Pressure‐induced swelling is previously found in MXene only under conditions of shear stress. Here clay‐like Ti_3_C_2_T_x_ MXene is synthesized from MAX phase by two different etching methods and compressed in diamond anvil cells (DAC) in liquid water and methanol. Pressure‐induced swelling is found only for MXene synthesized using LiF+HCl etching and only in liquid water but not in methanol.

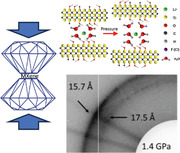

## 1 Introduction

Materials composed by 2D layers (2D materials) have attracted significant attention in recent years.^[^ ^1^ ^]^ In many applications, these materials are separated on single sheets to produce true 2D materials. However, significant interest was given also to multilayered 2D materials capable to swell in liquid solvents, for example, clays,^[^ ^2^ ^]^ graphene oxide (GO),^[^ ^3^ ^]^ and MXenes.^[^ ^4^ ^]^ The structure of these materials expands when exposed to vapors or liquid polar solvents due to intercalation of solvent molecules between 2D sheets. The expanded inter‐layer space of swollen structures is serving as “channels” for transport of solvents or dissolved molecules/ions in GO and MXene membranes.^[^ ^5^ ^]^ Ability to swell is also important in energy storage devices, e.g. for providing access to the surface of individual 2D sheets in MXene‐based electrodes for supercapacitors.^[^ ^6^ ^]^ Therefore, studies of MXene swelling under various environmental conditions (temperature and pressure) are of interest for both fundamental and applied science.

Studies of material's compressibility allow to obtain important information about the structure of stacked 2D materials in solvent‐free and solvent filled (solvated) states.^[^ ^7^ ^]^ Comparing compressibility of materials with “empty” (solvent‐free) and solvent filled inter‐layers allows to verify for possible effects of “pillaring” by intercalated molecules or possible interconnection between individual 2D sheets that would reduce the compressibility of the material in the direction perpendicular to the planes. Detailed studies of structural modifications and compressibility are available at the moment for several 2D materials,^[^ ^7^ ^]^ such as graphite and graphite‐like materials,^[^ ^8^ ^]^ h‐BN,^[^ ^9^ ^]^ natural and synthetic clays,^[^ ^10^ ^]^ graphite oxide,^[^ ^11^ ^]^ metal organic frameworks (MOF's) and covalent organic framewroks (COF's).^[^ ^12^ ^]^ The 2D materials typically show strong inter‐layer compressibility due to weak van der Waals bonding between the layers and rather low in‐plane compressibility.

One of the most unusual effects found in hydrophilic 2D materials is pressure‐induced swelling. Historically, intercalation of alcohols under GPa pressures was reported first for kaolinite^[^ ^13^ ^]^ and layered titanates.^[^ ^14^ ^]^ Reversible pressure‐induced swelling transitions were found later for GO in several polar solvents,^[^ ^11^, ^15^ ^]^ synthetic^[^ ^16^ ^]^ and natural clays.^[^ ^17^ ^]^ This effect is observed when hydrophilic 2D material is compressed in liquid solvent and undergoes swelling transition due to expansion of inter‐layer lattice caused by insertion of additional solvent, either as well defined layers or in “liquid‐like” state. Reversible swelling transitions between, for example, one‐ and two‐layered solvate phases were found in high‐pressure experiments with GO in alcohols^[^ ^15^, ^18^ ^]^ and synthetic hectorite in water.^[^ ^16^ ^]^

Similar swelling transitions were also considered as possible for Ti‐MXene, Ti_3_C_2_T_x_.^[^ ^19^ ^]^ This material consists of 2D sheets of titanium carbide Ti_3_C_2_ terminated by a variety of functional groups. Exactly what kind of functional groups terminate MXene depends on the synthesis method. The earliest reported synthesis of MXene uses HF acid for etching away aluminum from the MAX phase Ti_3_AlC_2_.^[^ ^6c^ ^]^ In this case, C‐F is likely to be the main type of functional group on MXene planes and the resulting material has rather limited swelling. “Clay‐like” MXene was later synthesized using HCL+LiF or LiCl+HF for etching Al from MAX phase.^[^ ^4^, ^20^ ^]^ This type of MXene is likely to exhibit more extensive functionalization with other groups (e.g., hydroxyls) which make it more hydrophilic. It absorbs water from air proportionally to humidity forming a randomly interstratified structure.^[^ ^21^ ^]^ Also, the presence of Li ions in the synthesis is likely to affect properties of MXenes making it more similar to certain types of clays with inter‐layer cations.^[^ ^4^, ^22^ ^]^ The clay‐like MXene was demonstrated to swell in several polar solvents^[^ ^4^, ^22^ ^]^ and to exhibit some swelling transitions under temperature variations.^[^ ^23^ ^]^

MXenes remain only rather scarcely characterized at extreme pressure (GPa) conditions.^[^ ^19^, ^24^ ^]^ High pressure studies of MXene swelling are so far limited only to one study by M.Ghidu et al.^[^ ^19^ ^]^ The samples synthesized using standard HF etching were found to swell in water with an increase of inter‐layer distance from 10 Å in water free state to 15 Å in liquid water. Compressing the MXene in liquid water did not result in pressure‐induced swelling transitions in experiments performed at pressures up to 3–5 GPa in Diamond Anvil Cells (DAC). Small increase of interlayer distance (compared to initial state) was found only after decompression.^[^ ^19^ ^]^ Notably, very few points were recorded in the pressure region below solidification of water in this study. Therefore, experimental data were sufficient to demonstrate absence of pressure‐induced swelling but not to evaluate compressibility along inter‐plane direction. Pressure‐induced swelling was found in this study only if shear stress is added to compression at relatively low pressures in MPa range with increase of d(001) from 12.5Å (MXene intercalated with one water layer) up to ≈15Å thus corresponding to insertion of second layer of water molecules.^[^ ^19^ ^]^

The study included also some experiments with MXene prepared using HCl+LiF etching but only at MPa range. This method of MXene synthesis is known to provide materials with “clay‐like” swelling. Therefore, if any Ti_3_C_2_T_x_ is capable to show pressure‐induced swelling transitions, this type of synthesis is the most likely candidate rather than HF type of MXene. Moreover, so far MXene swelling at GPa conditions was studied only in water but not in other solvents. It is known that GO shows well‐defined swelling transitions between differently solvated structures in alcohols and very different type of pressure‐induced swelling in water.^[^ ^15^, ^18^ ^]^ Therefore, there is strong interest to study MXenes for possible pressure‐induced swelling transitions in polar solvents other than water.

In this study, we have used DAC to compress MXenes synthesized using HCl+LiF or LiCl+ HF procedures in “dry” and liquid‐immersed state. We report first data for linear compressibility of MXenes in water and methanol in inter‐layer direction and compare it to other bulk materials composed by 2D sheets. The effect of pressure‐induced swelling was found for MXene only in water. The transition corresponds to insertion of one additional water layer into the MXene structure and formation of never previously reported three‐layered phase. However, the transition into the expanded phase was partial and observed only for MXene synthesized using HCl+LiF etching method.

## 2 Results and Discussion

**Figure 1:**
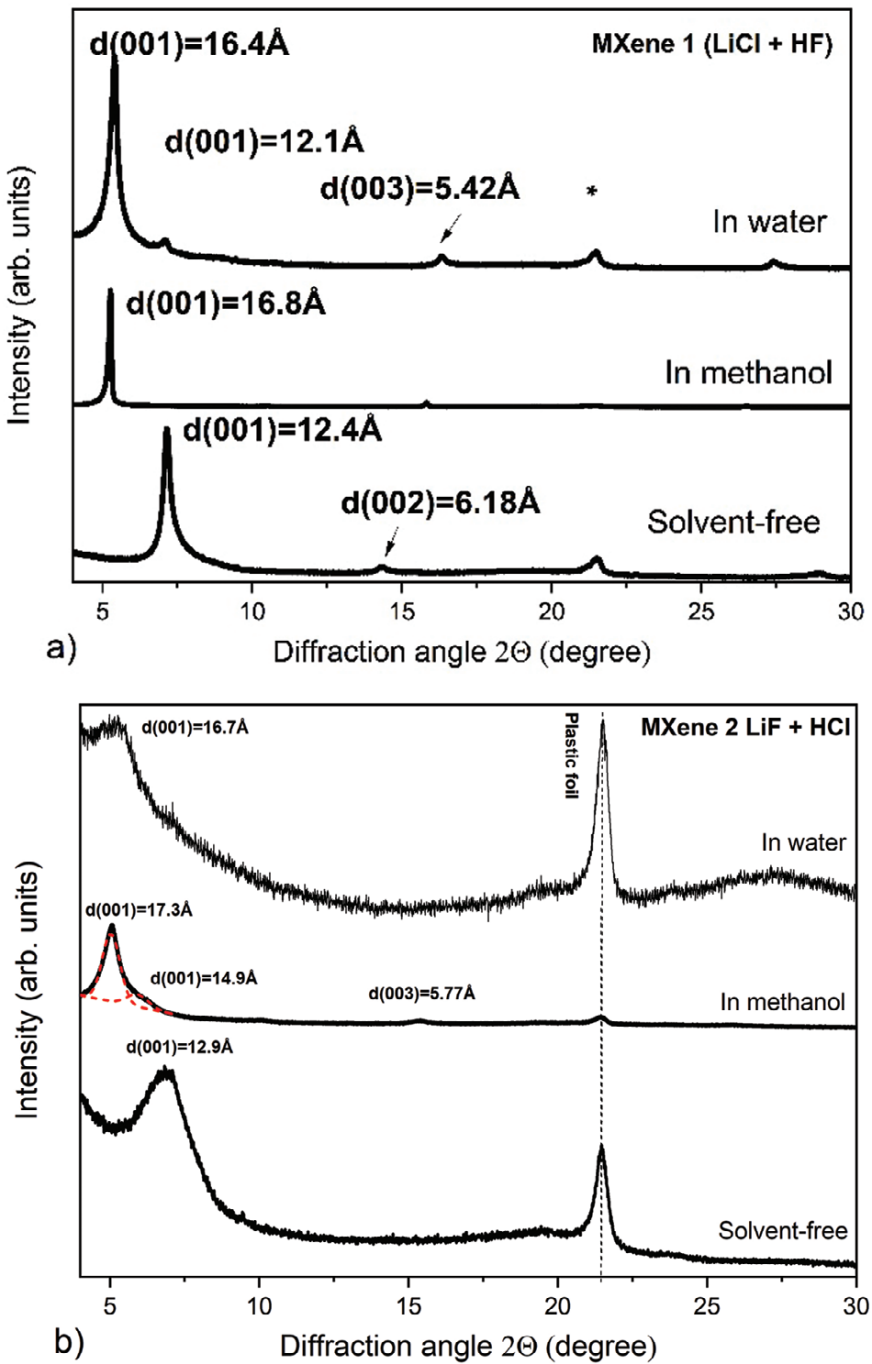
XRD patterns recorded for a) MXene 1 and b) MXene 2 in solvent‐free state, in methanol and water at ambient conditions using CuKα radiation. Dashed red line shows fitting using Voigt functions, which shows the presence of two reflections. Intensity of patterns recorded in methanol for both MXenes is divided by a factor of ten.

The swelling of freshly prepared MXenes was first analyzed at ambient conditions in liquid‐immersed state with methanol and water (**Figure** **1**). These data were used as a reference for high‐pressure experiments. Note that all experiments were performed within one week after the synthesis of new MXene batch, whereas the materials were stored under argon prior to loading into DAC in order to avoid effects related to material aging.

Interlayer distance of MXene structure was estimated using d(001). Note that we assume (001)‐reflection to correspond directly to inter‐layer distance rather than indexing it as (002) in P6_3_/mmc structure. There is no evidence of ordering of MXene layers in solvent‐free state and certainly not in MXene structures formed by swelling in polar solvents. The XRD patterns exhibited d(001) = 12.4 Å and d(001) = 12.9 Å for MXene 1 (LiCl+HF) and MXene 2 (LiF+HCl), respectively. These d‐spacings are in good agreement with literature data for MXenes intercalated with Li.^[^ ^4^, ^21^, ^22^, ^25^ ^]^ Analysis of XRD patterns recorded in liquid‐immersed state showed similar swelling for both MXenes in water and methanol (Figure 1) with increase of d(001) (compared to ambient state) by ≈4.4Å in methanol and ≈4 Å in water. Note also that MXene 1 showed sharper and stronger reflections compared to MXene 2. The intensity of XRD reflections recorded from MXene in methanol increases significantly compared to precursor solvent‐free sample (Figure 1). The increase of interlayer distance by 4.4 Å corresponds to the thickness of one methanol layer. The thickness of one water layer is expected to be ≈2.5 Å. Therefore, experimentally observed inter‐layer lattice expansion is closer to thickness of two water layers.

Note however, that the MXene structure shows no intercalation with discrete water layers and shifts gradually upon humidity change (at ambient pressure) due to interstratification.^[^ ^21^ ^]^ The structure of MXene in liquid water is also likely to be interstratified. It cannot be ruled out that the 4 Å increase corresponds to random interstratification of layers with different degree of hydration, for example, two layer (2L) state with a smaller fraction of one‐layer (1L) state.

**Figure 2:**
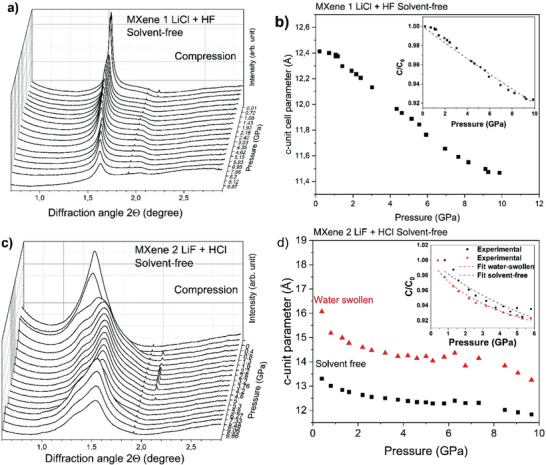
XRD patterns recorded from solvent‐free a) MXene 1 and c) MXene 2 at different pressure during compression. Pressure dependences of c‐unit cell parameter of b) MXene 1 and d) MXene 2. Fitting was performed for two (001) reflections: ■ – none swelling part ▴ – swelling part of samples. Fitting by Murnaghan equation was used to evaluate modulus in c‐direction (insets in b and d). XRD patterns of MXenes recorded during decompression are presented in Figures S8 and S9 (Supporting Information).

In situ high‐pressure X‐ray diffraction experiments were performed with powder samples of MXenes in diamond anvil cells (DAC) using synchrotron radiation in liquidimmersed state and in solvent‐free state (without any pressure medium) for reference. A sample of solvent‐free MXene prepared using LiCl+HF method (MXene 1) shows a single (001)‐reflection and almost linear pressure dependence for d(001) (**Figure** **2**a,b).

The compression experiment with MXene prepared using LiF+HCl method (MXene 2) showed less trivial results. This sample exhibited one rather broad but symmetric reflection with d = 14.2 Å. The pattern recorded at slightly higher pressure of 0.4 GPa exhibits two reflections with d‐spacings at 16.0 and 13.3 Å (at 0.4 GPa). This peculiarity we suggest to explain by partial hydration of loaded sample. The samples were loaded at ambient conditions absorbing water proportional to air humidity. Note that 14.2 Å value is slightly higher compared to 12.9 Å (Figure 1) recorded from bulk sample. We suggest that this increase is related to randomly interstratified hydrated and not hydrated MXene layers. However, the random interstratification is replaced at pressures above 0.4 GPa by segregated interstratification showing two reflections instead of one. We assume that the reflection found at 16.0 Å is related to water swollen part of the sample and second reflection (13.3 Å) to mostly water free part. Therefore, presence of two reflections allows to study compressibility of solvent‐free and water‐swollen states of MXene 2 simultaneously in one experiment (Figure 2c,d).

Linear compressibility was estimated from the change of c‐unit cell parameters (Figure 2b) using Murnaghan equation. Fitting provided modulus values of 105 GPa (Figure 2b) for MXene 1 and 50 GPa for MXene 2 (in inter‐layer direction) (Figure 2d). MXene 1 has an almost linear change of d‐spacing with pressure while MXene 2 shows clearly non‐linear dependence. We note that the in–plane compressibility of titanium carbide layers is rather small and cannot be reliably evaluated within the relatively narrow pressure range of our experiments. Therefore, we arranged the experimental setup to maximize the information about the small angle region, where the effects of pressure‐induced swelling were expected to appear (the region of the 001‐reflection).

**Figure 3:**
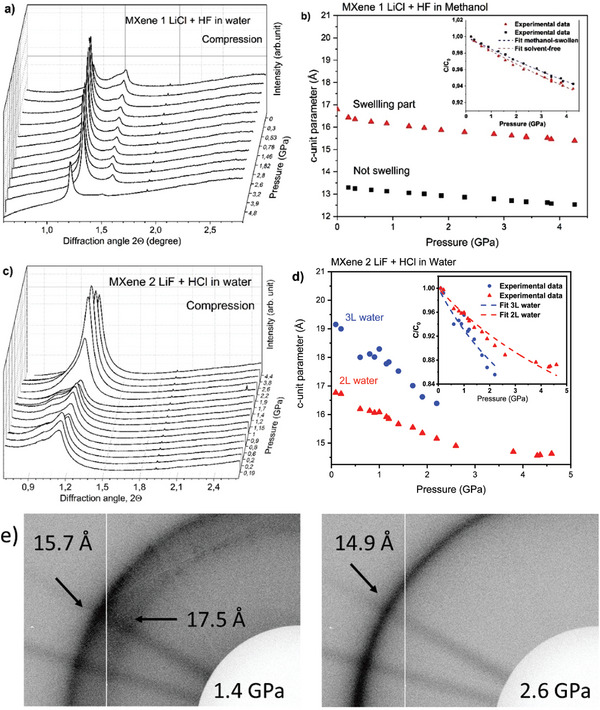
XRD patterns recorded from a) MXene 1 and c) MXene 2 in water during compression and pressure dependences of c‐unit cell parameter of b) MXene 1 and d) MXene 2. ■ – experimental data of solvent‐free MXene ▴ – experimental data of swelling MXene in water, ● – pressure‐induced swelling in water. Fitting by Murnaghan equation was used to evaluate modulus in c‐direction (insets in b and d); e) fragments of XRD images recorded below (1.4 GPa) and above (2.6 GPa) solidification point of liquid water medium. The patterns recorded in liquid water show presence of two phases with the difference in d‐spacing corresponding to one water layer (see Supporting Information file for second example).

Next, experiments were performed for samples of MXene 1 and MXene 2 immersed in water. The MXene 1 sample immersed in liquid water showed two (001)‐reflections: a weak one from the solvent‐free part and a strong reflection from the water‐swollen state (**Figure** **3**a) with the difference in d‐spacings of ≈4 Å. We assume that a smaller part of this sample has inter‐layers not accessible for water. One can see a clear difference in linear compressibility of MXene 1 estimated for these two states of the material (Figure 3b). The compressibility of MXene 1 calculated using d(001)‐spacing of the solvent‐free part of the pattern is 126 GPa, which is very similar to linear compressibility extracted from the experiment with solvent‐free MXene 1 (Figure 2a). A linear modulus of 55 GPa was found for the c‐direction of water swollen MXene 1. That is an almost two times lower modulus compared to solvent‐free MXene 1.

Therefore, the compressibility data support our initial assumptions for the origin of these two reflections from swollen and solvent‐free parts of sample. We note that MXenes are most often synthesized in slightly non‐homogeneous state. Compression of MXene 2 in water was recorded up to 4.6 GPa and showed rather different results. Two (001)‐reflections were found already at the lowest pressure point (0.2 GPa): a strong peak with d‐spacing 16.7 Å and shoulder corresponding to 19.2 Å (Figure 3c). Both values are significantly higher compared to the d‐spacing of solvent‐free MXene 2 (12.9 Å). The reflection with d‐spacing of 16.7 Å is most likely related to the intercalation of two water layer between MXene layers. This is in good agreement with the literature, where d‐spacing for the same reflection is reported in range 15.0–16.8 Å.^[^ ^4^, ^21^, ^22^, ^25^ ^]^ A similar d‐spacing 16.4 Å was observed also in XRD pattern recorded from precursor MXene 2 sample at ambient conditions. Note that additional shoulder was not found in the ambient pressure pattern (Figure 1). The shoulder with 19.2 Å d‐spacing is not an artefact as evidenced by observation of second diffraction ring in 2D XRD images. The relative intensity of (001)‐reflection from pressure‐induced swollen phase increases up to pressure of 1.4 GPa (Figure 3e). The two swollen phases of MXene also showed distinctly different pressure dependence of d(001) (Figure 3d). Existence of new pressure‐induced swollen phase of MXene 2 in water was also reproduced in one more experiment with new loading of material and pressure rapidly increased to ≈1.4 GPa to observe two diffraction rings from differently hydrated phases. Then the pressure was increased over the point of water solidification and released. This experiment confirmed results obtained in the experiment shown in the Figure 3.

Appearance of the new phase with expanded inter‐layer distance is interpreted as evidence for pressure‐induced swelling. The new phase is found already at the lowest pressure point (0.2 GPa). The water‐expanded high pressure phase disappears at pressures above the pressure point of water solidification ≈2 GPa (Figure 3d,e). Similar effect of water escape from expanded pressure‐induced swollen structure was observed previously for GO above the point of water solidification.^[^ ^11^ ^]^

The data suggest that MXene 2 sample is not exactly homogeneous with some part, which undergoes pressure‐induced swelling and other part remaining in pristine ambient pressure water‐swollen phase. The difference between d‐spacings of two swollen phases is ≈2.5 Å (19.2–16.7 Å at 0.2 GPa), which corresponds well to the size of water molecule. Assuming the same thickness for water layers, the ambient 16.7 Å phase should be related to intercalation of two water layers. In this case the reference inter‐layer distance of 11.7 Å should be found for MXene structure in completely solvent‐free state. The value 11.7 Å is somewhat smaller compared to the 12.9 Å value recorded in our ambient conditions XRD. However, it was recorded at ambient humidity and d(001) could be slightly expanded due to sorption of water in randomly interstratified hydrated/non hydrated stacks. Note also that 19.2 Å value was recorded under 0.2 GPa pressure and will be slightly higher if extrapolated to ambient pressure. Therefore, we consider our data to be in good agreement with three‐layer structure of pressure‐induced swollen phase of MXene 2. In following discussions, we will name ambient MXene‐water phase as 2L (two layers of water) and pressure‐induced phase as 3L (three layers of water).

The ≈2.5Å thickness for water layers confined between 2D sheets was found previously in other hydrophilic materials, for example GO^[^ ^26^ ^]^ and clays.^[^ ^16^ ^]^

**Figure 4:**
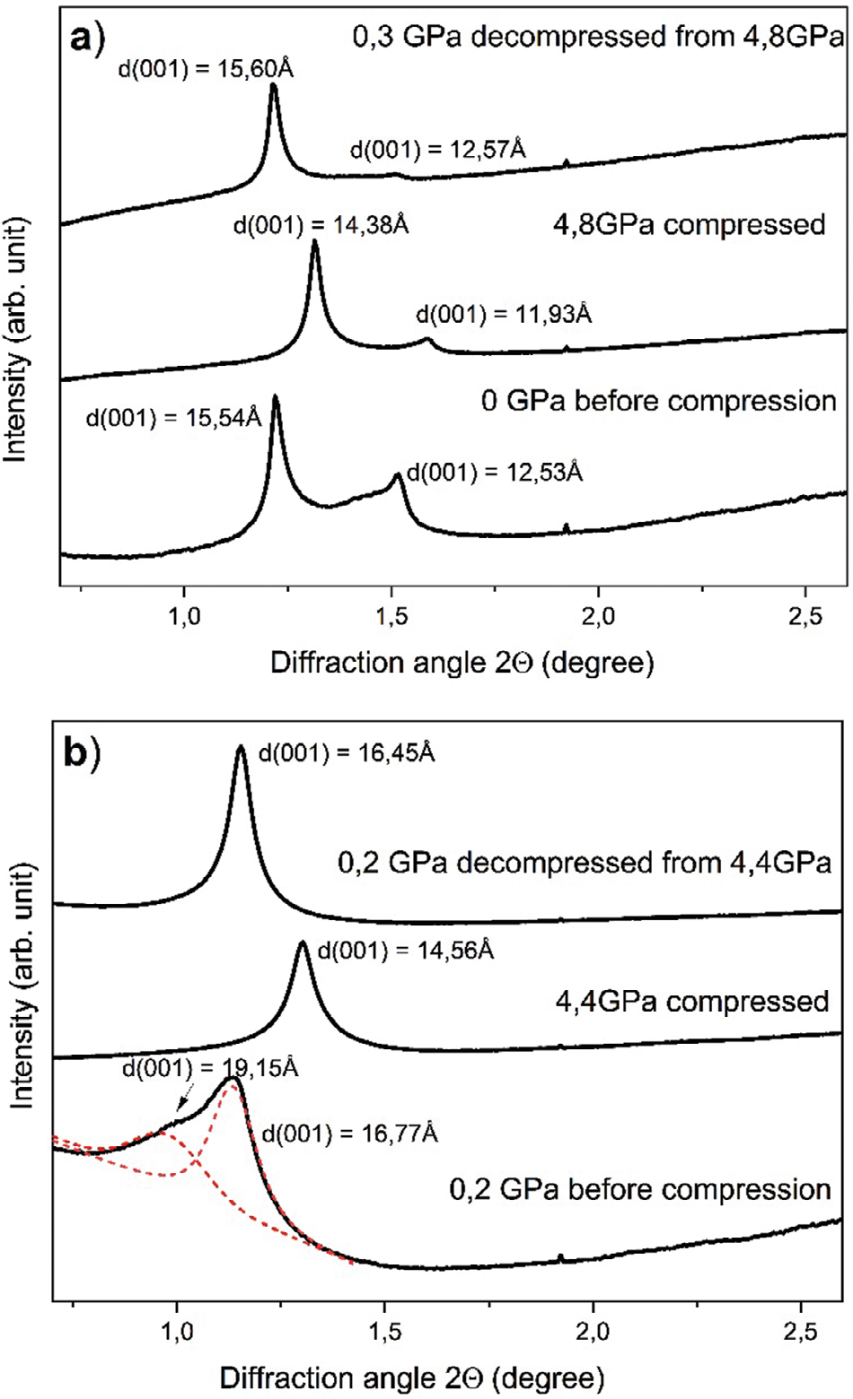
XRD patterns recorded from a) MXene 1 and b) MXene 2 at first pressure point, at pressure above water solidification point and after decompression.

It can be concluded that pressure‐induced swelling does occur in our experiment with MXene 2 but only for part of the sample and at pressures below 0.2 GPa. The pressure‐induced swollen phase is stable in equilibrium with liquid water medium but disappears after water solidification. The pressure‐induced phase does not re‐appears after pressure release (**Figure** **4**b, see Supporting Information file for complete decompression data). The XRD reflection assigned to pressure‐induced swollen MXene 2 is not found after decompression

Therefore, it can be concluded that the pressure‐induced swelling transition is reversible but occurs at rather low (for DAC experiments) pressures in the range 0–0.2 GPa.

Experiments with compression of MXene 1 and MXene 2 were performed also with methanol‐immersed samples. However, pressure‐induced swelling was not found in these experiments.

**Figure 5:**
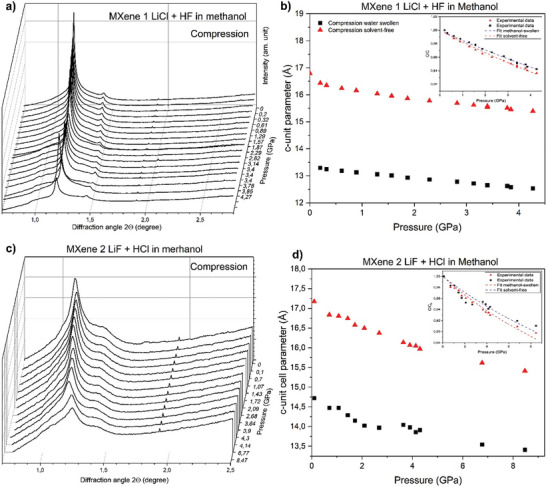
XRD patterns recorded from a) MXene 1 and c) MXene 2 in methanol at different pressures during compression and pressure dependences of c‐unit cell parameter of b) MXene 1 and d) MXene 2. ■ – experimental data of solvent‐free MXene ▴ – experimental data of swelling MXene in methanol. Insets show experimental compressibility of graphite oxide in c‐direction with fitting by Murnaghan equation. Patterns of MXenes recorded during decompression are presented in Figures S12 and S13 (Supporting Information).

XRD patterns of MXene 1 recorded in methanol at low pressure showed two peaks. The first reflection with a d‐spacing of 16.8 Å is strong and sharp and corresponds to the “ambient” swollen phase of MXene (**Figure** **5**a). The second peak has weak intensity and corresponds to a d‐spacing of 13.3 Å that probably correspond to solvent‐free MXene. Note that the difference of 3.5 Å is in good agreement with experimentally observed thickness of one methanol layer intercalated in GO structure.^[^ ^18^ ^]^ Patterns recorded in the DAC at low pressure are in good agreement with the data recorded using conventional XRD from powder sample at ambient conditions (Figure 1). The experiment with MXene 1 in methanol was extended up to pressures of 9.14 GPa. However, the quality of patterns decreased dramatically after methanol solidification (≈3.86 GPa, Figure S7, Supporting Information) and did not recover after decompression. XRD patterns of MXene 2 also showed two peaks at low pressure (Figure 5c). The strongest reflection corresponds to phase with d‐spacing 17.2 Å, which is in good agreement with the data recorded under ambient conditions (Figure 1). The second weak reflection corresponds to d‐spacing 14.7 Å somewhat higher than the value found for solvent‐free MXene. A weak shoulder reflection with similar d‐spacing can be found in a pattern recorded at ambient conditions (Figure 1). Compression of both MXene 1 and MXene 2 does not reveal any effects that could be assigned to pressure‐induced swelling in methanol.

Linear compressibility of methanol‐immersed MXenes in the inter‐layer direction was estimated from the dependence of the c‐unit cell parameter versus pressure. For MXene 1, only the data points up to 4.3 GPa were used due to much lower quality of XRD patterns recorded above the pressure of methanol solidification (Figure 5b). At the same time, in the experiment with MXene 2 in methanol the data points up to 8.5 GPa were possible to record with sufficiently good quality (Figure 5c,d). The data are summarized in **Table** **1**.

**Table 1:** Modulus in c‐direction (inter‐layer direction) determined in compression experiments with two types of MXenes. In case if two (001) reflections were found, fitting of both components was performed. The d(001)‐values are given for these components at the lowest pressure point (≈0.2 GPa). The (001) reflections were assigned to phase hydrated/solvated with different number of water layers (0L‐ not hydrated, 1L‐ one layer, 2L‐two layers).

Type of MXene	Fitted (001) reflections: d‐spacing and number of solvent layers.*	Modulus in c‐direction (GPa)
MXenes Solvent‐free		
MXene 1	12.4 Å (0L)	105
MXene 2	13.3 Å (0L)	50
MXene 2	16.0 Å (2L)	26
MXenes in water		
MXene 1	12.5 Å (0L)	126
MXene 1	15.5 Å (2L)	55
MXene 2	16.7 Å (2L)	21
MXene 2	19.1 Å (3L)	12
MXenes in methanol		
MXene 1	13.3 Å (0L)	63
MXene 1	16.4 Å (1L)	55
MXene 2	14.7 Å (0L)	64
MXene 2	17.2 Å (1L)	54

The data characterizing linear compressibility of solvent‐free and solvent swollen MXenes 1 and 2 are summarized in Table 1.

Several observations can be done by analysis of the Table 1 data.

- Compressibility of swollen phases is significantly stronger compared to “solvent‐free” MXene. This result could be anticipated considering van der Waals bonding between individual MXene sheets.
- The distance between neighboring MXene sheets is significantly larger in swollen phases that results in much weaker interaction mediated through solvent molecules. The modulus value is even smaller for pressure‐induced 3L phase in agreement with larger MXene sheets separation.
- There is also a significant difference in compressibility of MXene 1 and MXene 2 materials. MXene 2 is in general more compressible and shows smaller modulus values for the same types of structures (0L, 1L, and 2L). We note that the samples were loaded under ambient humidity. Therefore, the samples absorbed some water from air and were partly hydrated.

The data suggest that MXene 2 sample had stronger swelling not only under compression but also included more water at the moment of loading. The sample loaded on air included some part that was hydrated by sorption of moisture from air but this part showed nearly the same compressibility as the sample hydrated in liquid water. Note that modulus values of (2L) phases of MXene 2 are very similar for samples loaded with and without liquid water (21 and 26 GPa, respectively). Therefore, significantly higher modulus value of MXene 1 and lower compressibility can be explained by absence of hydrated part (no sorption of water form air). Similar results were obtained for MXene 1 and MXene 2 samples compressed in methanol. The part of sample solvated with 1L of methanol is also more compressible compared to 0L phase, in agreement with the general trend.

It is interesting to compare compressibility's of MXenes in c‐direction with other materials composed by 2D sheets and van der Waals bonding between layers. The c‐direction modulus of “dry” MXenes (105 and 50 GPa) is significantly higher compared to other 2D materials (e.g., 1.4 GPa for GO^[^ ^11^ ^]^). However, the value of inter‐layer modulus does not directly reflects the changes for distance between 2D layers as it is related to C_0_ unit cell parameter which is almost twice larger in MXene structure composed by relatively thick titanium carbide layers. Comparing the distance between 2D sheets and absolute values for changes of inter‐layer distance is more meaningful if we are to compare the change of volume between 2D sheets. Obviously, the compressibility of Ti_3_C_2_ layers in c‐direction must be negligibly small compared to change in inter‐layer distance. The change of inter‐layer distance found for MXene 1 and MXene 2 is similar to other layered materials with van der Waals bonding between the layers. For example, the change of interlayer distance after compression to 5 GPa is ≈0.7Å for solvent‐free MXene 1 and also ≈0.7Å for GO at the same pressure point (Figure 2b).

Possibly the most interesting result of our study is the first observation of the pressure‐induced swelling in MXene synthesized using LiF+HCl etching method and additionally lithiated in LiCl solution (MXene 2). Swelling of hydrophilic 2D materials is known to be affected very strongly by the presence of interlayer cations. For example, clay minerals are known to show swelling related to hydration of interlayer cations. It is also not unusual for clays to exhibit crystalline swelling with precisely defined step‐like transitions between phases intercalated with different numbers of water layers.^[^ ^27^ ^]^ Clays intercalated with cations are known to swell in water and at least in some cases the size of cations determines a precisely defined number of inserted water layers.^[^ ^28^ ^]^

Therefore, Li cations are likely to be a factor helping not only the swelling of MXene at ambient conditions but also assisting in pressure‐induced swelling. XPS data show that Li‐content is similar for both MXenes (7.1 at% for MXene 2 and 7.4% for MXene 1 respectively). However, XPS does not provide information about how homogenous is Li intercalation. It is known that homogeneity of charge distribution and charge compensation cations is one of the important conditions for crystalline swelling of clays.^[^ ^29^ ^]^ The MXene etched using only HF (without Li‐salts) was studied earlier at GPa pressures but pressure‐induced swelling was not found in these experiments.^[^ ^19^ ^]^ Intercalation of Li cations is known to make swelling stronger prompting to name this type of MXenes as “clay‐like”.^[^ ^20^ ^]^

Notably, the swelling under conditions of shear was found in earlier study also for MXene synthesized using Li‐ salts.^[^ ^19^ ^]^

The difference in swelling properties of MXene 1 and MXene 2 can also possibly be related to somewhat higher abundance of hydrophilic functional groups attached to titanium carbide sheets. However, exact analysis of relative amounts of different functional groups is difficult due to overlaps in characteristic peak positions of these groups in XPS spectra. Therefore, smaller oxygen content detected in MXene 1 by XPS (16.7 at% compared to 20.8 at% in MXene 2) (see also Figure.S1 and S2 in SI file) could be a key factor which inhibits pressure‐induced swelling in MXene 1. Swelling of MXene can also be affected by ageing effects. MXenes are known to degrade when exposed to humid air, in our experience especially strongly if stored as a powder. Further experiments are required to verify detailed mechanism and structure features of MXene enabling or disabling pressure‐induced swelling.

The incomplete transition into pressure‐induced 3L phase is likely explained by inhomogeneous nature of MXene. The structure consists of negatively charged layers and charge neutrality is warranted by interlayer Li‐ cations. Only if the charge density and thus the concentration of interlayer cations is homogeneous, the swelling will be uniform for all layers in the material. We speculate that the etching procedure leaves part of inter‐layers incompletely accessible for lithiation while effects of interstratification^[^ ^21^ ^]^ complicate distinguishing differently attached and expanded by swelling layers.

It should be emphasized that the pressure‐induced 3L phase is completely novel and has never been reported in earlier studies. The only available earlier study of pressure‐induced swelling in Ti_3_C_2_T_x_ demonstrated a transition from a 1L – water phase to a 2 – water phase with a change in d(001) by 2.5 Å for each inserted water layer (**Table** **2**). The MXene materials studied here demonstrated 2L‐phases being immersed in liquid water already at ambient pressure. The formation of the 3L‐phase with d(001) = 19.2 Å under conditions of hydrostatic compression in liquid water is completely new effect.

**Table 2:** Summary of literature data for pressure‐induced swelling of 2D materials in water medium. The columns (from right to left): name of material and synthesis details; increase of inter‐layer distance due to swelling at ambient pressure conditions Δd(001); change of inter‐layer distance due to pressure‐induced swelling Δd^*^(001); overall increase of d(001) which includes both ambient and pressure‐induced swelling Δd(001) max; presence or absence of literature data for pressure‐induced swelling in solvents other than water; references.

Material	Δd(001) swelling ambient	Δd*(001) pressure‐induced	Δd(001) Max	Other solv.	References.
MXene Ti_3_C_2_T_x_ (LiF+HCl)	3.7 Å (2L)	2.4 Å (0.2 GPa)	6.3 Å (3L)	no	This study
MXene Ti_3_C_2_T_x_ (HF)	5 Å (2L)	none	5 Å (2L)	no	[19]
MXene Ti_3_C_2_T_x_ (HF+LiCl)	2.5 Å (1L)	2.5 Å (0.3 GPa, uniaxial)	5 Å (2L)	no	[19]
Na‐Hect	5 Å (2L)	2 Å	6.5 Å (3L)	no	[16]
Kaolinite	0	3 Å (2.5 GPa)	3 Å (1L)	yes	[17]
Graphite oxide	5 Å	3 Å (1.6 GPa) 11 Å (2.0 GPa, in NaOH)	8 Å 16 Å* (Interstr.)	yes	[11, 15]

We suggest that the “natural” water‐immersed state of MXene (in Li‐intercalated state) corresponds to the 2L structure. However, if the Mxene has some defects connecting 2D layers in some points (e.g., due to incomplete etching of all Al atoms or due to effects of ageing), the swelling can be limited to only 1L –intercalation or result in the complete absence of swelling. Shear stress helps to slide 2D layers relative to each other to break few defects connecting interlayers. The materials used in our study were synthesized shortly (1 week) prior to high pressure experiments and loading into DAC in order to minimize effects of ageing. An alternative explanation of better hydration of our samples at ambient conditions could be more complete and homogeneous Li‐intercalation. Following initial synthesis, the materials were kept in 1 m LiCl solution for 24 h under argon to ensure saturated intercalation of Li.

It is interesting to compare MXene to other materials exhibiting pressure‐induced swelling in polar solvents (Table 2) in more detail. So far, only GO demonstrated pressure‐induced swelling with continuous increase of interlayer distance upon compression in liquid water. The increase of GO interlayer distance Δd(001)≈3Å is highest just below the pressure point of water solidification corresponding approximately to the insertion of one water layer.^[^ ^11^ ^]^ The increase of interlayer distance due to pressure‐induced swelling is even stronger in NaOH solutions Δd(001)≈11Å (at 1.6 GPa) and in this case can be considered as an osmotic type of swelling.

The osmotic type of pressure‐induced swelling remains to be unique for GO and only for compression in basic water solutions. Crystalline pressure‐induced swelling corresponding to a step‐like change of interlayer distance due to the insertion/de‐insertion of an additional layer is found for GO in other solvents (methanol, ethanol, acetone). Crystalline pressure‐induced swelling is also found in all other materials in liquid water, and  in few other polar solvents . Pressure‐induced swelling in layered titanates, some synthetic and natural clays occurs in a step‐like transition with an increase of interlayer distance corresponding to the insertion of one water layer (2–2.5Å). Once the additional water layer is inserted at certain pressure point, further increase of pressure results in “standard” compressibility expected for the new phase in absence of solvent migration between solid and liquid phases. Our study demonstrates that linear compressibility in c‐direction is stronger for phases hydrated with larger number of water layers and significantly stronger compared to solvent‐free state of MXene.

The data shown in Table 1 confirm that pressure‐induced swelling typically results in the insertion of one additional layer of water for all 2D materials. This type of transition either occurs in one step (crystalline swelling) or as a continuous change from one state to another that is commonly explained by effects of interstratification.^[^ ^3^, ^30^ ^]^ Nevertheless, the swelling of interstratified layers is likely to be crystalline, that is corresponding to exact number of inserted water layers

It is still an open question why some hydrophilic materials exhibit pressure‐induced swelling while other materials not. For example, montmorillonite exhibits swelling at ambient conditions but experiments has not revealed pressure‐induced hydration at GPa pressures.^[^ ^31^ ^]^

Rich variety of pressure‐induced effects found in GO is likely related to the fact that 2D layers of this material are composed by light elements (carbon and oxygen) and are relatively thin. Therefore, swelling pressures developed in process of GO hydration^[^ ^32^ ^]^ are sufficient for the expansion of interlayers even for solvents other than water. MXene structure is composed by relatively thick layers and includes heavy Ti atoms which might explain at least partly why pressure‐induced swelling was not found for methanol‐immersed samples. Alternatively, the role of intercalated cations present in MXene and absent in GO structure could be rather significant. For example, hydration of fluorohectorites is known to depend significantly on the size and charge of intercalated cations^[^ ^28c^ ^]^ but effects of cation size on swelling transitions were never studied at GPa pressure. Swelling of MXenes with different exchange cations was also studied only at ambient pressure.^[^ ^4a^ ^]^

For future outlook, it is interesting to study pressure‐induced swelling for MXenes in a broader variety of polar solvents and using materials prepared with intercalated cations other than Li.

## 3 Conclusion

In conclusion, “clay‐like” MXenes synthesized using etching with LiCl+HF and HCl+LiF were compressed in “dry” state, in water and in methanol above the point of liquid solvent solidification. Pressure‐induced swelling in liquid water was found for MXene synthesized using HCl+LiF method already at relatively low pressure of 0.2 GPa but not for MXene prepared using HCl+HF method. The change of interlayer distance due to the pressure‐induced swelling corresponds to a transition from a 2L‐water MXene phase into a 3L‐water phase. This new high‐pressure phase Ti_3_C_2_T_x_ with inter‐layer distance of 19.1 Å (at 0.2 GPa) is novel and never observed at ambient pressures. The 3L‐phase is not preserved above the pressure point of water solidification and is not recovered after decompression. Compression in methanol revealed no anomalies related to insertion or de‐insertion of solvent into or from MXene structure. The modulus for inter‐layer c‐direction of MXene in solvated/hydrated states was found to be approximately twice smaller than in solvent‐free state. Moreover, compressibility of 3L water MXene is twice higher compared to 2L water MXene for c‐direction. Pressure‐induced swelling has been observed previously only for compression of MXene in water at MPa pressures under strong shear. Therefore, Li‐intercalated MXene is a new member in the family of hydrophilic 2D materials that exhibit pressure‐induced swelling in addition to layered titanates, graphite oxide, natural, and synthetic clays.

## Conflict of Interest

The authors declare no conflict of interest.

## Supporting information

Supporting Information

## Data Availability

The data that support the findings of this study are available from the corresponding author upon reasonable request.
