# Supplementary material for: Swelling of Ti3C2Tx MXene in Water and Methanol at Extreme Pressure Conditions
Source: Adv Sci (Weinh). 2023 Dec 14;11(9):2307067. doi: 10.1002/advs.202307067 (PMC10916643; doi:10.1002/advs.202307067)
Supplement: Supplementary file 1 — Supporting Information [file ADVS-11-2307067-s001.pdf]

ADVANCED SCIENCE
Open Access

Supporting Information

for *Adv. Sci.*, DOI 10.1002/advs.202307067

Swelling of $Ti_3C_2T_x$ MXene in Water and Methanol at Extreme Pressure Conditions

Artem Iakunkov, Ulrich Lienert, Jinhua Sun and Alexandr V. Talyzin**

Supporting information

Swelling of $Ti_3C_2T_x$ MXene in water and methanol at extreme pressure conditions.

Artem Iakunkov, [a] Ulrich Lienert,[b] Jinhua Sun[c]* and A. V. Talyzin1*[a]

[a] Department of Physics, Umeå University, Umeå, SE-901 87, Sweden. alexandr.talyzin@umu.se

[b] DESY Photon Science, 22607 Hamburg, Germany

[c] Department of Industrial and Materials Science, Chalmers University of Technology, SE-412 96 Göteborg, Sweden. jinhua@chalmers.se

Supporting information for this article is given via a link at the end of the document.

1. Materials and methods.
2. Details of high pressure experiments.
3. Additional data for high pressure experiments showing XRD patterns recorded during decompression.

1. Material and methods:

$Ti_3C_2T_x$ synthesis

$Ti_3C_2T_x$ samples were synthesized using two procedures reported previously. [41-44] First method includes etching of precursor Ti_3AlC_2 MAX phase (®Carbon, Ukraine) with a mixture LiCl (99% Sigma Aldrich) and 10% HF (diluting commercial 48% HF by Sigma Aldrich) for 22h at 50°C (MXene 1) [41, 42]. The second procedure involves treatment with a mixture of LiF (98% Sigma Aldrich) and HCl (37% Sigma Aldrich) for 22h at 40ºC (MXene 2). Typically, a molar ratio 1 to 5 between initial MAX phase and LiCl/F was used for both procedures. After the end etching procedure, resulting mixtures were centrifuged (typically used 15 min 15000rpm) and washed with 6M HCl in order to remove all Li-salt impurities. The resulting material was placed in 1M LiCl solution for 24h under argon to ensure saturated intercalation of Li in final structure[41]. After this, material was washed using water until pH 6 is achieved. Finally, the water dispersion is centrifuged using 500-1000 rpm for 10 min and only supernatant is collected and vacuum dried to obtain MXene.

Characterization of materials after synthesis.

XPS spectra were recorded with a Kratos Axis Ultra electron spectrometer equipped with a delay line detector. A monochromatic Al Kα source operated at 150 W, a hybrid lens system with a magnetic lens, providing an analysis area of 0.3 × 0.7 mm, and a charge neutralizer were used for the measurements. The binding energy scale was adjusted with respect to the C 1s line of aliphatic carbon, set at 285.0 eV. All spectra were processed with the Kratos software[45](see **Figure S1-2** in SI) Panalytical X'pert X-ray diffractometer with Cu-Ka radiation was used to record the diffraction patterns at ambient conditions immediately after synthesis (see **Figure S3** in SI).

High-pressure experiments

High-pressure experiments were performed in four posts diamond anvil cells (DAC) using diamonds with culet sizes of 0.3-0.6 mm. The samples were loaded into a 0.2-0.3 mm hole in

the steel or rhenium gaskets together with a ruby chip and a piece of gold wire used for pressure calibration. The pressure was increased gradually in steps of 0.1-1.5 GPa, and XRD patterns were recorded on every step during compression and decompression.

XRD patterns were recorded from MXene samples using synchrotron radiation at DESY Germany, beamline P21.2 (λ= 0.330670 Å) using a Perkin Elmer XRD1621 area detector to record patterns, covering d-spacing range from 33.5Å to 6.3Å and two Varex XRD4343CT area detectors to record pattern, covering d-spacing range from 3.1Å to 1.0Å. The low angle region covered by first detector is ideal for recording reflections related to MXene interlayer distance while two other detectors at low angle region were used to record data for gold pressure calibrant. The detectors were calibrated using AgBeh calibrant. The two-dimensional XRD patterns were integrated using pyFAI and Dioptas 3.1 software. More details can be found in SI.

The one-dimensional analogue of the Murnaghan equation of state[18, 46] provides an approximation for describing the nonlinear relation between normalized lattice parameters and pressure(eq. 1):

$$\frac{r}{r_0} = \left[1 + \left(\frac{\beta'}{\beta}\right)^{1/\beta'}\right] \quad (1)$$

Here, r is the lattice constant along one of the crystal axes, $\beta = -\left(\frac{dP}{dlnr}\right)_{P=0}$ is the linear compressibility, and β' is the pressure derivative of β. Linear compressibility of MXene was calculated using Eq.1 with parameters obtained from a least-squares fit to the experimental data. β' was fixed in the range from 1 to 4.

Characterization of materials. XPS data.

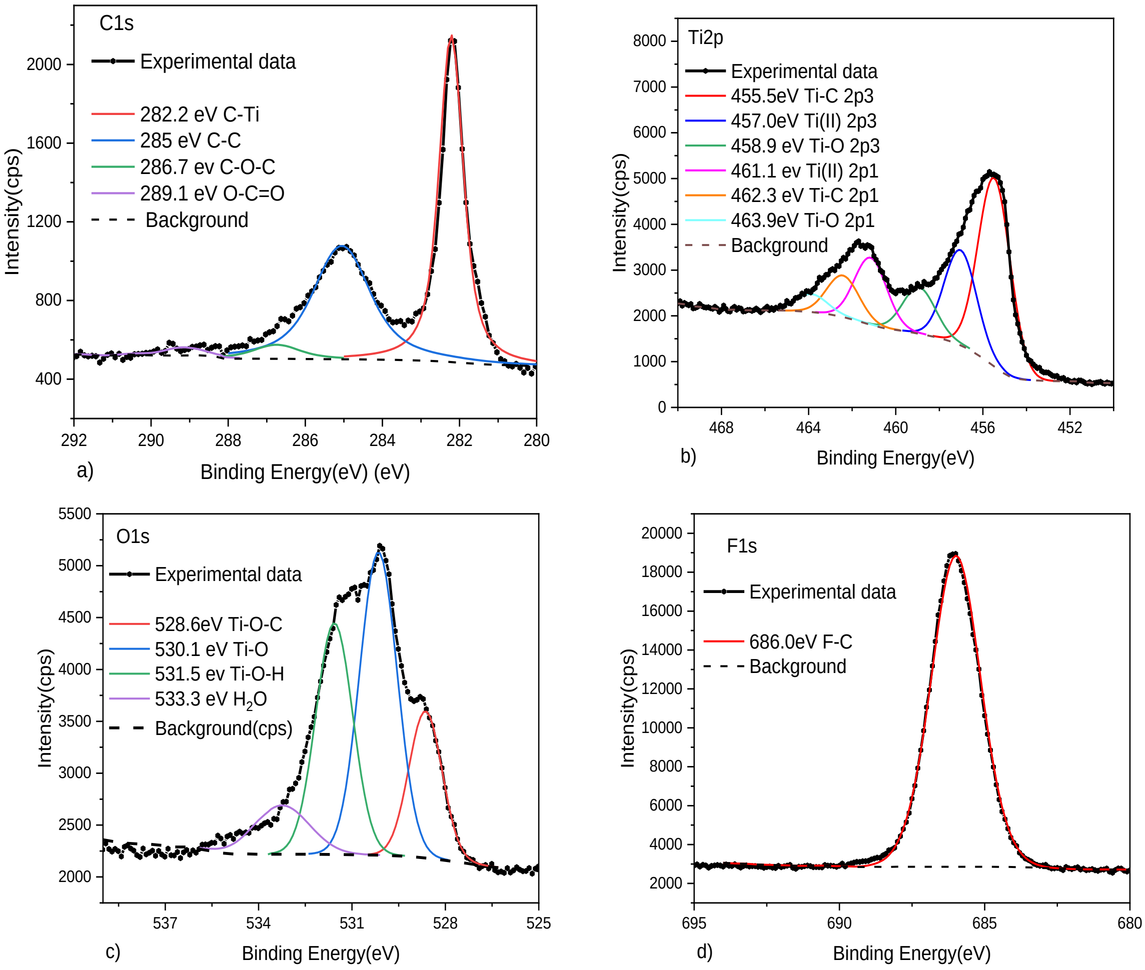

Figure S1. X-ray photoelectron spectra of MXene prepared using LiCl+HF method: a) C1s ; b) Ti2p; c) O1s; d) F1s.

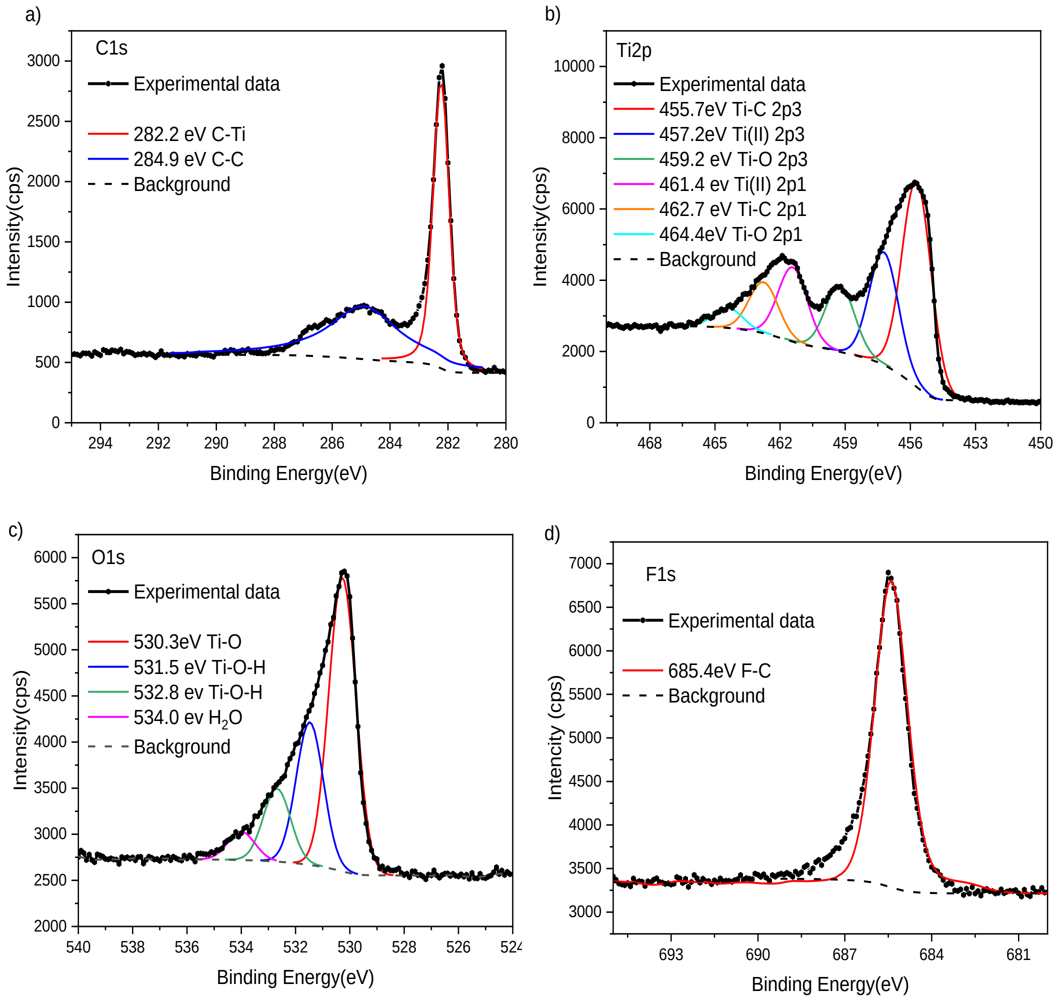

Figure S2. X-ray photoelectron spectra of MXene prepared using LiF+HCl a) C1s; b) Ti2p; c) O1s; d) F1s

XRD at ambient conditions

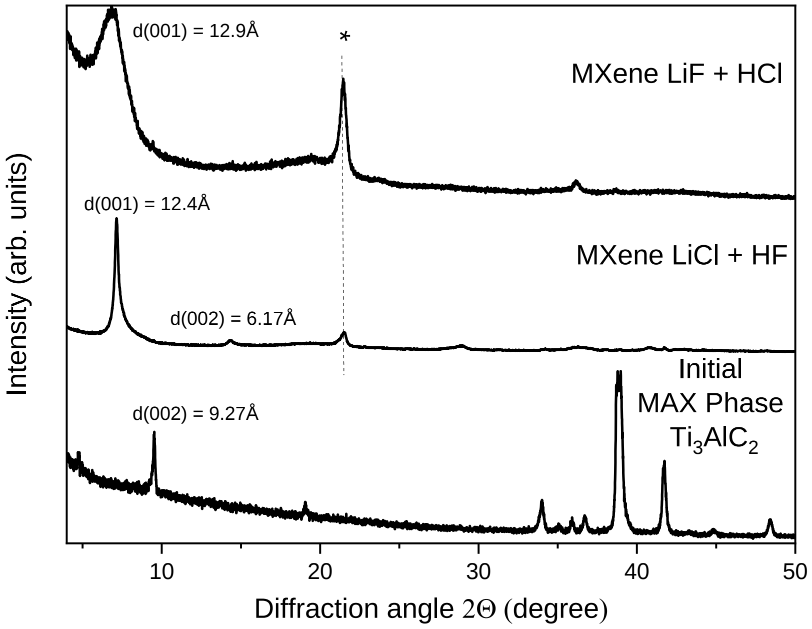

Figure S3. XRD pattern of initial MAX phase and MXene synthesized using LiCl + HF and LiF + HCl etchings, CuKα radiation.

High pressure experiments

Four post Diamond anvil cells (DAC) with culet size of diamonds 0.3-0.6 mm were used. The samples were loaded into a 0.2-0.3 mm hole in the steel or rhenium gaskets (**Figure S5b**) together with a ruby chip and piece of gold wire used for pressure calibration. The pressure was increased gradually in steps of 0.1-1.5 GPa, and XRD patterns were recorded on every step during compression and decompression.

XRD patterns were recorded in DAC's using synchrotron radiation at DESY Germany, beamlines P21.2 (λ= 0.330670 Å) using a Perkin Elmer XRD1621 area detector to record pattern, covering d-spacing range from 33.5Å to 6.3Å and two Varex XRD4343CT area detectors to record pattern, covering d-spacing range from 3.1Å to 1.0Å **(Figure 6S)**. The detectors were calibrated using AgBeh calibrant.

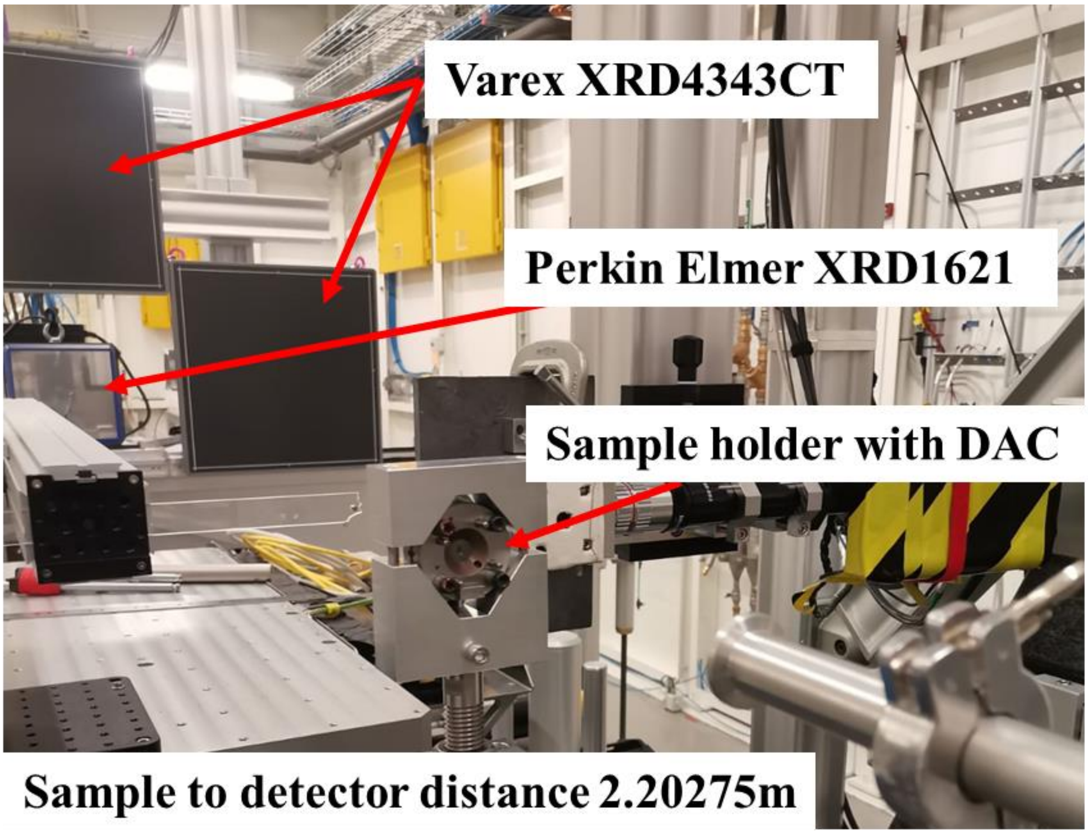

Figure S4. Image from P21.2 beamline (DESY Hamburg Germany). Here one can see typical setup for high pressure measurement with DAC in sample holder and two types of detectors to record signal in two different angle ranges.

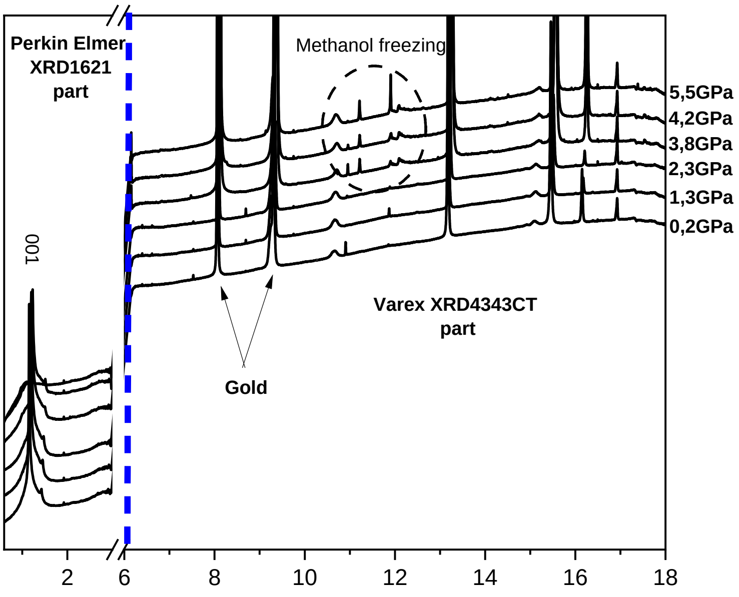

Figure S5. Patterns of MXene 1 in methanol recorded at different pressures using using two detectors

Additional data for high pressure experiments with MXenes. XRD patterns recorded during decompression.

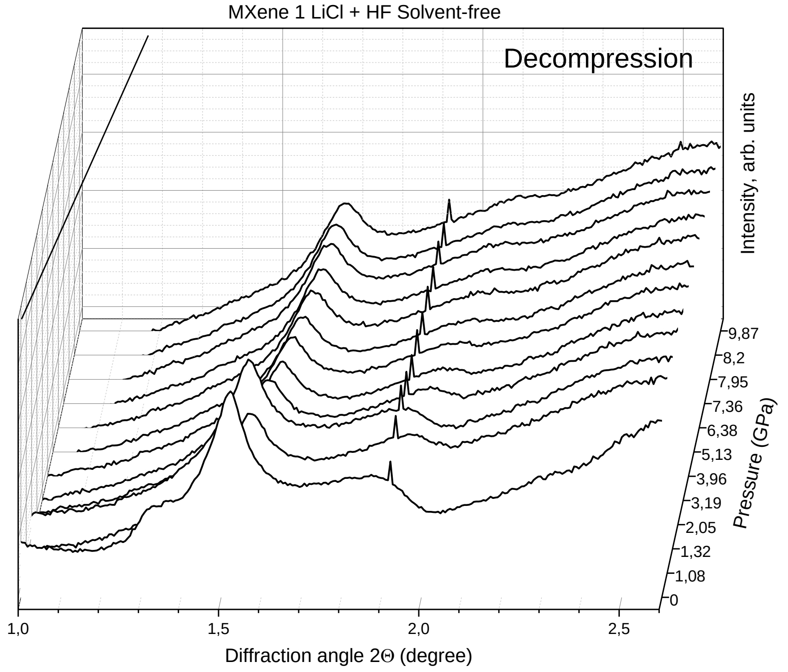

Figure S6. XRD patterns recorded from solvent-free MXene 1 at different pressure up to 9.87GPa; during decompression

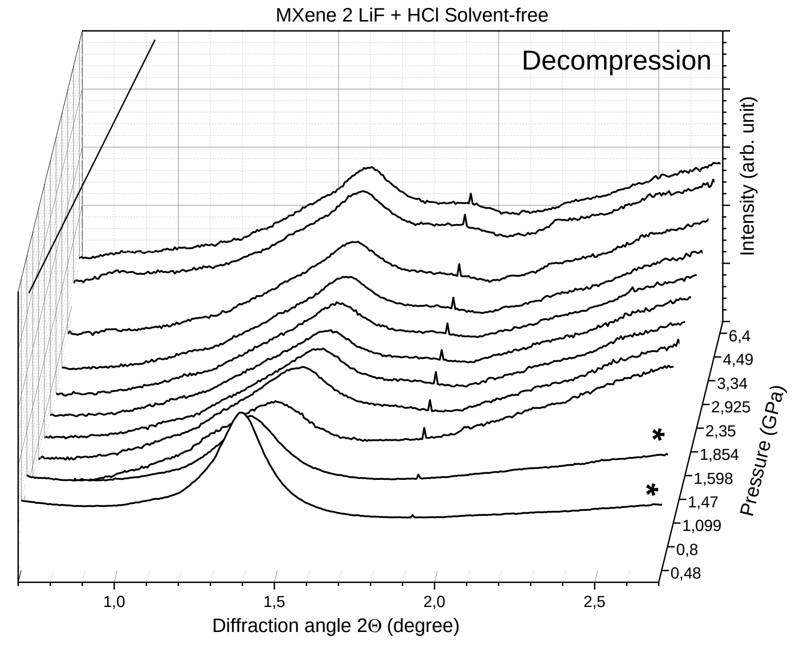

Figure S7. XRD patterns recorded from solvent-free MXene 2 at different pressure upto 9.66GPa; during decompression. Intensity of patterns marked with * was divided by factors 3 and 10 respectively for better representation.

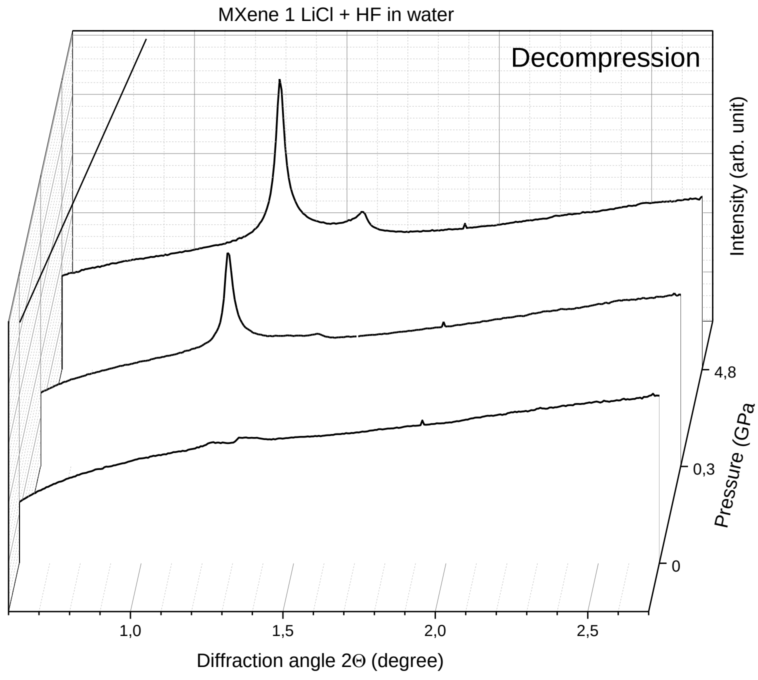

Figure S8. XRD patterns recorded from MXene 1 in water during decompression at different pressure. Experiment was recorded up to 4.8 GPa with fast decompression.

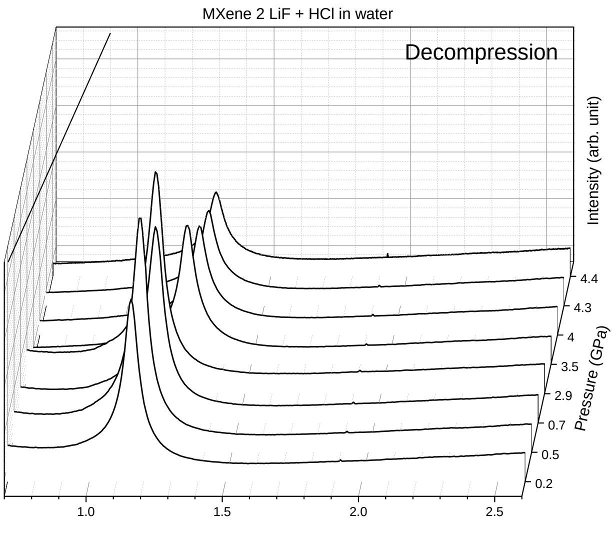

Figure S9. XRD patterns recorded from MXene 2 in water during decompression at different pressure.

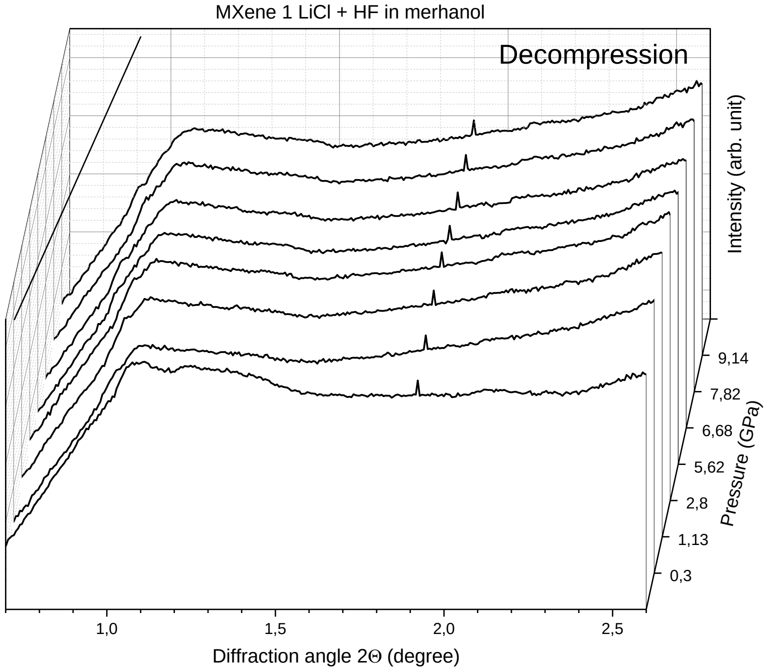

Figure S10. XRD patterns recorded from MXene 1 in methanol during decompression at different pressure. Experiment was recorded up to 9.14GPa. Quality of pattern decreased dramatically after methanol freezing and did not recover after decompression.

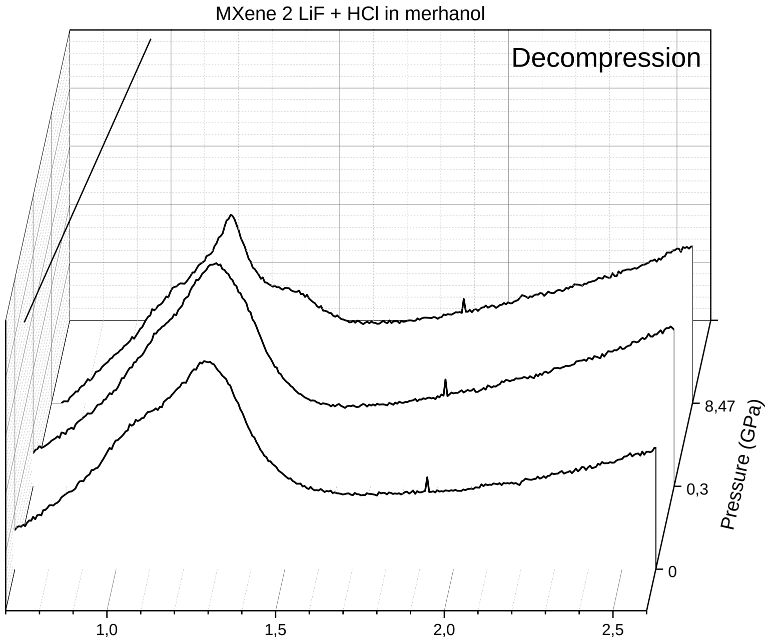

Figure S11. XRD patterns recorded from MXene 2 in methanol during decompression. Experiment was performed with detailed recording of data up to 8.47GPa with fast decompression in few steps.

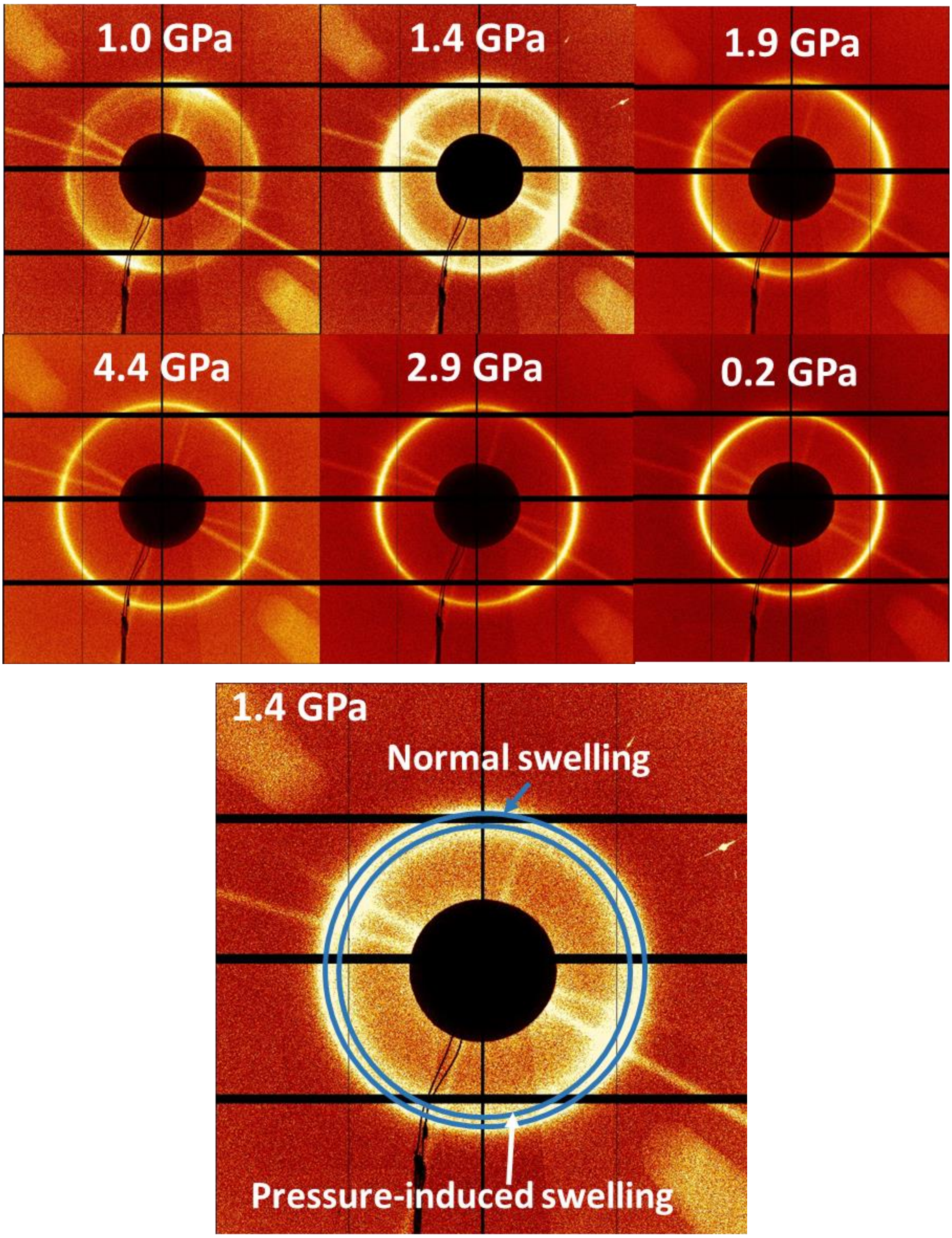

Figure S12. 2D images recorded for MXene 2 in water during at different pressure. 2D image recorded at 1.4GPa has two rings indicating presence of reflection generated by pressure-induced swelling
